# Women’s Management of Recurrent Bacterial Vaginosis and Experiences of Clinical Care: A Qualitative Study

**DOI:** 10.1371/journal.pone.0151794

**Published:** 2016-03-24

**Authors:** Jade Bilardi, Sandra Walker, Ruth McNair, Julie Mooney-Somers, Meredith Temple-Smith, Clare Bellhouse, Christopher Fairley, Marcus Chen, Catriona Bradshaw

**Affiliations:** 1 Central Clinical School, Monash University, Melbourne, Victoria, Australia; 2 Melbourne Sexual Health Centre, The Alfred Hospital, Melbourne, Victoria, Australia; 3 Department of General Practice, The University of Melbourne, Melbourne, Victoria, Australia; 4 Centre for Values, Ethics and the Law in Medicine, The University of Sydney, Sydney, New South Wales, Australia; 5 Melbourne School of Population and Global Health, The University of Melbourne, Melbourne, Victoria, Australia; University of Illinois at Urbana-Champaign, UNITED STATES

## Abstract

**Background:**

Few data are available on how women manage recurring bacterial vaginosis (BV) and their experiences of the clinical care of this condition. This study aimed to explore women’s recurrent BV management approaches and clinical care experiences, with a view to informing and improving the clinical management of BV.

**Methods:**

A descriptive, social constructionist approach was chosen as the framework for the study. Thirty-five women of varying sexual orientation who had experienced recurrent BV in the past 5 years took part in semi-structured interviews.

**Results:**

The majority of women reported frustration and dissatisfaction with current treatment regimens and low levels of satisfaction with the clinical management of BV. Overall, women disliked taking antibiotics regularly, commonly experienced adverse side effects from treatment and felt frustrated at having symptoms recur quite quickly after treatment. Issues in clinical care included inconsistency in advice, misdiagnosis and inappropriate diagnostic approaches and insensitive or dismissive attitudes. Women were more inclined to report positive clinical experiences with sexual health physicians than primary care providers. Women’s frustrations led most to try their own self-help remedies and lifestyle modifications in an attempt to treat symptoms and prevent recurrences, including well-known risk practices such as douching.

**Conclusion:**

In the face of considerable uncertainty about the cause of BV, high rates of recurrence, unacceptable treatment options and often insensitive and inconsistent clinical management, women are trying their own self-help remedies and lifestyle modifications to prevent recurrences, often with little effect. Clinical management of BV could be improved through the use of standardised diagnostic approaches, increased sensitivity and understanding of the impact of BV, and the provision of evidence based advice about known BV related risk factors.

## Introduction

Bacterial vaginosis (BV) is the most common vaginal condition affecting women of reproductive age, with prevalence estimates of between 10–30% among women who have sex with men in developed nations [1, 2] and 20–50% of women who have sex with women (WSW) [3–7]. The symptoms of BV include an abnormal malodour and increased discharge [8, 9]. Past studies have found the symptoms of BV to be highly distressing and embarrassing to women, impacting significantly on their self-esteem and sexual relationships and making them feel ‘dirty’, embarrassed, ashamed, self-conscious and fearful others will detect their abnormal odour or discharge [10]. Adverse sequelae associated with BV includes miscarriage, preterm delivery and increased risk of sexually transmitted infections (STIs) and HIV [11–13].

Unfortunately, the aetiology and whether BV is sexually transmitted remains unknown and current treatment options are associated with recurrence rates in excess of 50% within 12 months of treatment [14]. Recommended first line treatment for BV includes oral metronidazole or topical clindamycin cream and while effective in the short term, adverse side-affects are common and include nausea, vomiting, an unpleasant taste in the mouth and vaginal candidiasis [14, 15] and symptom relief is often short lived. It remains unclear whether recurrence reflects reinfection or persistent infection [14, 16]. Current treatment guidelines do not define BV as an STI or recommend partner treatment [17] and women are generally informed by clinicians BV is not an STI.

In an attempt to treat or prevent recurring vaginal symptoms women will often employ their own self-help remedies such as douching, taking yoghurt orally or vaginally, probiotics or vitamin supplements, using over the counter yeast infection treatment products and antiseptic creams, wearing cotton underwear and avoiding hot baths and perfumed soaps [18–24]. Most of these self-help remedies however, have little effect, with studies consistently showing douching to be associated with a higher incidence of BV [1, 25–28]. Data on the efficacy of alternative treatments for BV are generally of poor quality, however there is limited evidence to suggest probiotics, lactic acid based treatments and antiseptics may offer some benefit in the treatment of BV [24, 29, 30].

While BV is the most common vaginal infection among women, few studies have explored in-detail women’s experiences of the clinical management of BV. Past research has shown women often feel dissatisfied with the clinical care they receive when presenting with vaginal symptoms [18, 21] and both patients and clinicians frequently misdiagnose BV [20, 23, 31–34]. This study aimed to explore women’s recurrent BV management approaches and clinical care experiences, with a view to informing and improving the clinical management of BV.

## Methods

Detailed methods for this study have been outlined in an earlier paper [10]. This study has been reported in accordance with the Consolidated criteria for reporting qualitative research (COREQ) guidelines [35].

### Ethics statement

Ethical approval for this study was granted by the Alfred Hospital Ethics Committee, Victoria, Australia, Application Number 318/12 on the 23^rd^ October 2012.

### Synopsis of Methods

A social constructionist approach was chosen as the framework for the study. Semi-structured interviews were chosen as they allowed the opportunity for women to tell their lived experiences and personal realities of recurrent BV while also allowing for the exploration of key clinical areas of interest. To be eligible for the study women had to be aged 18 to 45 years, have experienced two or more diagnosed episodes of BV in the past five years and have a good understanding of verbal and written English. Women were purposively sampled to allow for a broad sample of women including heterosexual and WSW, single women and women in a relationship, women who had experienced high and low numbers of recurrent BV and women from a number of recruitment locations. Women were recruited from the Melbourne Sexual Health Centre (MSHC), the largest sexual health clinic in Victoria, Australia; a previous longitudinal BV study of Australian WSW (The WOW Health Study) [36]; and specialist sexual health medical clinics or general practices with a high case load of female patients of reproductive age (hereafter referred to as high caseload clinics). Participants had the option of being interviewed either by telephone or face to face at MSHC or in their own home. Participants interviewed face to face at MSHC were provided with a written plain language statement (PLS) and consent form to read and sign. Participants interviewed by telephone were read aloud the PLS and consent form and asked to provide verbal consent. Verbal consent was obtained for telephone interviews as it was not practical to obtain written consent for this method of interview. Verbal consent was recorded on the consent form by way of the researcher signing on the participant’s behalf and a copy forwarded by post for their records. This process of written and verbal consent was approved by the Alfred Hospital Ethics Committee.

After women had provided informed consent they were asked a series of 15 structured demographic, sexual behaviour and diagnosis and treatment questions before being asked questions pertaining to their knowledge of BV prior to their first episode, their first and recurrent experiences of BV, the impact of BV on them emotionally, socially, sexually and in their work lives, their beliefs around the causes and triggers of BV, their use of self-help remedies and their experience of antibiotic treatment and the clinical management of BV. Findings relating to the impact of BV on women emotionally, socially, sexually and in their work lives and on women’s experiences of the triggers of BV onset and exacerbating factors for recurrence are reported in previous papers [10, 37]. All interviews were conducted by JB or SW between November 2012 and January 2013. Thematic analysis [38] was undertaken and data coded using primarily a segmented approach [39]. Transcripts were imported into N-Vivo 9 for data management and a subset of transcripts reviewed independently by two other research team members to cross check coding and themes (MTS, SW). Analyses of demographic, sexual behaviour and diagnosis and treatment data were conducted using SPSS 20.0.

## Results

A total of 35 women participated in the study. Table 1 outlines participant demographics.

**Table 1:** Recruitment site and participant characteristics (demographic, sexual behaviour, diagnosis and symptoms of BV) N = 35.

	N or Median [Range]
**Recruitment site**	
MSHC	22
Longitudinal BV study	7
High caseload clinic	6
**Age**	30 [21–43]
**Born in Australia**	21
**Education level**	
Secondary school	5
TAFE diploma or certificate	9
Undergraduate degree	14
Post graduate certificate or degree	7
**Employment status**	
Full time	11
Part time	7
Casual	2
Student/Student & part time work	11
Unemployed	4
**Sexual Identity**	
Heterosexual	19
Lesbian	7
Queer	3
Bisexual	4
Other (pansexual/transgender)	2
**Sex industry worker^**	
No	29
Yes	6
**Smoke cigarettes**	
No	23
Yes	10
Past smoker	2
**Regular relationship**	
No	14
Yes	21
**Sex of partner**	
Male	13
Female	8
**Number of male sexual partners <5 years (if > = 1)**	10 [1–1300]
**Number of female sexual partners <5 years (if > = 1)**	3 [1–45]
**Number of times had BV in the past**	4 [2–25]
**Number of times had BV diagnosed in the past**	3 [2–25]
Symptoms*	
Abnormal odour	34
Abnormal discharge	35
**Most distressing symptom**	
Abnormal odour	30
Abnormal discharge	7

### Knowledge of BV

Overall, women reported very poor levels of awareness about BV prior to first diagnosis with most reporting they had never heard of BV before.

*I think nobody knows anything about it*, *like I had never even heard of it until I had it*. *I had never read anything or heard about talk about t or you know*, *seen it anywhere*. *So nobody really knows*. *I think unless you’ve had it yourself you wouldn’t be aware of what it is (Participant 15*, *age 37)*.

Some women attributed the lack of public knowledge or awareness of BV to societal stigma and shame women feel around STI’s and vaginal conditions.

*… it’s quite isolating*, *because women don’t talk about it*, *and there must be*, *must be women in my life who have had it and I mean we just don’t talk about it–it’s that shame I suppose*, *embarrassment…I wonder if I didn’t talk to very many people about it because I think it was triggered by having sex with other people and that’s not something that I really*, *we really talk to anyone else about…And it’s all caught up in that whole fear of being judged (Participant 7*, *age 39)*

Women were more likely to have heard of BV if they worked in the health industry, sex industry, attended sexual health clinics or participated in the longitudinal BV study.

### Women’s BV management approaches

#### Confusing BV for thrush

Women commonly thought they had thrush when they experienced BV for the first time and treated themselves for such. When symptoms persisted, women then often feared they had an STI or something ‘*more sinister’* and sought medical assistance. Consequently, many women did not seek medical assistance for up to a few weeks after symptoms first presented, however, sought treatment sooner with subsequent episodes.

*… it was quite distressing because initially I*…*what I thought was the thrush didn’t clear up*, *well nothing changed after I took that treatment*, *I thought maybe I had a STI (Participant 7*, *age 39)*.

Women who immediately feared their symptoms of BV were indicative of an STI rather than thrush were more likely to seek medical assistance quickly.

…*that’s why I went to the sexual health clinic ‘cause I was worried about having a STD (Participant 13*, *age 22)*

#### Women’s views and experiences of antibiotic treatment for BV

In general, most women felt that current clinical treatment options for BV were extremely limited, often ineffective and unacceptable in the long term. Women commonly experienced adverse side effects from antibiotic medication including nausea, stomach cramps, diarrhoea, thrush and a metallic taste in the mouth. Despite seven day antibiotic treatment generally being effective at the time of treatment, many women felt frustrated and distressed at having BV recur, often quite quickly after treatment.

*…it just became really clear that the symptoms were back and the smell was back*, *and I just thought ‘What am I going to do*?*’ and it was just such a short time*, *and it’s not insignificant treatment*, *you know*, *a week of Flagyl is pretty full on…But it was pretty distressing that it came back so quickly (Participant 29*, *age 26)*.

A couple of women felt their BV had never really gone away despite receiving treatment while others reported having episodes spanning over several months, the symptoms of which could be exacerbated by various factors [37] and the severity of which would influence whether they sought medical treatment or tried to self-manage symptoms.

Most women disliked taking antibiotics, especially on a regular basis, and felt frustrated at the lack of alternative effective treatment and preventative options.

*I do feel like it’s not a very good way of dealing with something recurring*, *that’s so recurring*, *like just to have antibiotics every time it’s recurring*. *Like there’s gotta be something else (Participant 1*, *age 25)*.

Consequently, a handful of women either no longer or rarely used antibiotics to treat BV either because they disliked taking repeated antibiotic treatment, could not tolerate the side effects or would rather use alternative therapies unless it was a symptomatic or severe episode.

#### Women’s use of self-help remedies to treat symptoms of BV

Women’s embarrassment around BV symptoms, their frustration and dislike of repeated antibiotic treatment and their preference for natural alternatives meant the majority of women had tried some form/s of self-help remedy to try and treat symptoms of BV. Self-help remedies included the use of alternative treatments such as probiotics, douching, over the counter vaginal treatments and various ‘home remedies’. Home remedies included salt or vinegar baths, the internal or external use of yoghurt or garlic and inserting tampons soaked in various products. Women also commonly reported having frequent showers, changing their underwear, using sanitary products and perfumes or deodorants to try and mask the symptoms of BV. Approximately a quarter of women had not tried any self-help remedies as they did not think they would be useful, feared they would make the symptoms worse or knew they were not recommended in the treatment of BV. Table 2 provides examples of the self-help remedies women tried to treat symptoms of BV.

**Table 2:** Women’s use of self-help remedies to treat the symptoms of BV.

**Douching**	.* *.* *.*I used that vinegar*, *that little barrel she [nurse] gave me again and with a bit of vinegar and water and that seemed to work (Participant 19*, *age 34)*.* *.* *.* *.*hydrogen peroxide I use more*, *I think maybe it’s a little bit stronger and better at killing everything off*. *And cos it’s liquid it kind of moves into all the corners really easily and that just worked really well*. *I think I only used it once or twice and it had completely gone*.* *.* *.*(Participant 10*, *age 23)*
**Baths**	*I just sort of like tried different homeopathic things like the bath where you sit in apple cider vinegar diluted with water*. *That kind of managed the symptoms and then it kind of went away (Participant 28*, *age 29)*.
**Suppositories**	*I tried it [garlic] both ways I tried eating it*, *I tried using it as like an internal thing*. *And it did help a bit but it didn’t make it go away*, *just lessened the symptoms (Participant 29*, *age 26)*. *I tried a tampon with salt*, *like organic salt and organic cream as well*. *It worked a little bit but antibiotics worked better (Participant 9*, *age 28)*.
**Over the counter vaginal treatments**	*I’ve started using the Acid Gel [pH balancing vaginal gel] sometimes after like some of the times I’ve had lots of penetrative sex*, *it seems to stop it sort of flaring up so much (Participant 14*, *age 40)*.
**Alternative medicine**	*It was just like Blackmore’s bio-balance [vitamin]and it just said take one a day or two a day when it’s needed so I took two a day for a week and it was fine*.* *.* *.*I guess like it didn’t go away completely it just kinda helped (Participant 27*, *age 21)*.*… seeing a women’s health naturopath is what’s done it for me*. *I think it was Golden Seal [herbal supplement]*. *Yep capsules every night for a month*, *and then I went back to see her*, *it took a couple of weeks before I really noticed any reduction in symptoms*, *I didn’t use anything else in that time*.* *.* *.*(Participant 7*, *age 39)*.

#### Success of self-help remedies

Some women reported their self-help remedies were very helpful and these women were more likely to feel in control of their BV because they felt they were able to treat it somewhat effectively themselves

*…Yeah*, *when I got it again that was something I did straight away*, *immediately [used hydrogen peroxide]*. *I didn’t have to mess around with other things (Participant 10*, *age 23)*.

Most women however reported their self-help remedies did not help, and for some simply exacerbated their symptoms.

*…I feel dirty so I wash myself a lot so I feel clean*. *So the more I wash myself*, *the worse it gets (Participant 30*, *age 31)*.

#### Women’s lifestyle modifications to prevent recurrences

In addition to self-help remedies to treat the symptoms of BV, many women had made longer term sexual and non-sexual lifestyle modifications in an attempt to prevent further recurrences. Women commonly tried to improve their diet, reduce alcohol intake and increase their amount of exercise either because they felt these factors may have been contributing to their susceptibility to BV or simply to see if the changes would help. Other non-sexual lifestyle changes included wearing cotton underwear only, avoiding tight clothing, avoiding soaps or using soap free washes and improving their general hygiene practices. A number of women also changed some sexual practices and behaviours they felt may be contributing to or triggering episodes including improving levels of sexual hygiene, no longer sharing sex toys and minimising the exchange of bodily fluids. Single women, who blamed themselves for acquiring BV through sex with casual partners, were most likely to report reconsidering their future perusal of casual sexual partners.

…*there’s an element making me a little bit reluctant to go looking for it [casual sex] again just cos*, *because it seems to be associated with a lot of hassle and side effects*…*it definitely makes me think about how casual sex*, *like it puts everything in a different light because it comes with the whole sort of consequences*… *(Participant 11*, *age 32)*.

Approximately one third of women had not implemented any long term lifestyle modifications as they felt there was little point given there was no evidence to suggest they would be useful in preventing recurrences. Table 3 provides examples of women’s lifestyle modifications.

**Table 3:** Women’s lifestyle modifications to prevent recurrences of BV.

Lifestyle modifications to prevent recurrences	
**Changes in sexual practices & hygiene**	.* *.* *.*I make sure nothing goes anywhere near the area like I’m so conchi [sic ‘conscientious’] about*.* *.* *.*nothings touching that unless it’s fully washed and clean and it’s supposed to be there (Participant 5*, *age 38)*…* *.*we don’t play like that anymore*, *so if we’ve mucked around with her*, *we wash hands and*, *yeah*, *I don’t want to risk it (Participant 6*, *31)*.
**Personal products & clothing**	.* *.* *.*[I] use soap free washes*, *wear loose clothing*, *obviously no nylon underwear or anything like that (Participant 3*, *age 39)*. *I try not to use fabric softeners or soaps*, *the toilet paper that I use is hypoallergenic*. *And so I try not to wear tight clothes or sweaty undies or anything (Participant 30*, *age 31)*.
**Improved health and wellbeing**	.* *.* *.*I’m going into a really raw diet so getting rid of all the sugar from my diet and looking into what they pump into produce*. *So I have really changed my diet since last November (Participant 32*, *age 43)*…* *.*I used to eat rubbish food like just*, *like I’ll live on noodles and things that aren’t really healthy*. *So I make sure I go shopping every three days [now] and I get fruits and vegetables and I make sure that you know*, *everything is healthy*. *(Participant 5*, *age 38)*.

#### Success of lifestyle modifications

A few women, who strongly believed they knew what triggered their BV, reported no further episodes since implementing these lifestyle changes however these women were more likely to report their regular female sexual partner had been treated for BV or they had changed sexual partners.

*…she didn’t have the symptoms that I did*, *so she didn’t realise she had it*. *So then when I encouraged her to go and do the study [WOW Health Study] to find out and then when she found out she had it she was treated and then that’s when we haven’t had it since (Participant 4*, *age 40)*.

For most women, long term lifestyle modifications had little effect on recurrences, resulting in a strong sense of frustration and for some women a sense they would always have BV.

…*not knowing how to get rid of it*, *I try to eat correctly so that my diet was good for that*, *try to wear not tight clothing*, *keep everything dry*, *I try everything to try to prevent it and it happens (Participant 29*, *age 26)*.

### Women’s experiences of the clinical management of BV

Overall, many women felt frustrated by clinician’s–in particular primary care providers—lack of, and inconsistency of knowledge about BV, their sometimes poor and insensitive management of BV and their dismissal or lack of understanding of the impact of recurrent BV. Women who had attended clinics specialising in sexual health issues tended to have higher levels of satisfaction with clinician’s levels of knowledge and support and felt more comfortable discussing BV with clinicians specialising in the area rather than primary care providers. Table 4 provides examples of women’s experiences of the clinical management of BV.

**Table 4:** Women’s experiences of the clinical management of BV.

Primary care	
**Insensitive or dismissive attitudes**	..*the woman I saw [the doctor]*, *as soon as I started asking questions*, *you know like*, *‘Well what can I do? What can I do to get rid of BV?’ she was very condescending*, *and you know…it wasn’t serious enough to treat me like someone with a real problem*. *And any questions*, *you know*, *sort of that I brought up*, *were just sort of palmed off (Participant 15*, *age 37)*. *…with BV they regularly dismiss it and go*, *“Oh that’s not an issue*, *it’s not a problem*, *it just goes away by itself” (Participant 3*, *age 39)*.
**Misdiagnosis and inappropriate diagnostic approaches**	*…she’s [doctor] like ‘Yeah*, *it’s thrush’ so treated the thrush and the symptoms didn’t really go away*. *So I went back to the doctor and she tested me and it was BV’ (Participant 6*, *age 31)*…* *.*I said [to my GP]‘I’ve got this odd discharge*, *I’d like a sexual health screen’ and so he ordered my tests…he didn’t test for BV*. *And at the same time I wanted a PAP test so I went to Family Planning thinking that would be the best place for me to go and get a PAP test*.* *.* *.*and I mentioned to them as well and they said ‘Well it sounds like BV*, *lets test for that’*. *And that’s the first really that I knew*, *started to know what it was (Participant 7*, *age 39)*.
**Inappropriate, inconsistent or no advice**	*…in general I think the information and discussion around it is quite vague and varies place to place*. *And that there’s never any solid opinion about it… I think that’s particularly important when you’re advising a patient about their behaviour and not really having any idea what*. *Like people giving you varying information because they don’t actually know*. *Like I’d rather you say*, *‘I don’t actually know the answer to that question’ than tell me something that you’re not really sure is true or that is inconclusive and not really taking steps to make sure that’s clear (Participant 22*, *age 28)*.
**Sexual health services**	
**Higher satisfaction levels with sexual health services**	*So the people there are pretty good with sexual health stuff overall so I felt comfortable talking about it…I’ve spoken to two of them about it*, *one of them said*, *‘No one knows much about BV’*, *the other one said*, *‘We know a lot about BV but we don’t know what to do with it (laughs) the vagina’s a very complex environment’…so I feel like they’re interested in it and know something I ‘spose (Participant 14*, *age 40)*. *…to be honest*, *I really wouldn’t see a GP for this kind of thing*. *I feel far more comfortable coming in and seeing someone who this is their job*, *and they care about it (Participant 21*, *age 27)*…* *.*it’s just there isn’t enough facts or explanation of it*. *I felt that the [sexual health centre] was very interested and wanted to find out what’s going on with BV*. *So I found that very comforting that someone was interested (Participant 29*, *age 26)*.

It was not uncommon for women to report primary care providers had often confused BV for thrush with a number stating they had been diagnosed and treated for thrush without further testing or tested and/or treated for chlamydia or other STIs without being tested for BV. A couple of women also recounted seeing primary care providers who would not prescribe antibiotic treatment for BV because of adverse side effects or because they were told ‘*it would just resolve itself’*. A number of women described feeling frustrated at the differing and inconsistent advice they received from clinicians in general about BV including it: is ‘*just a woman’s thing’*, is associated with how sexually active you are, is an imbalance of bacteria, it could be transmitted by uncircumcised men, is similar to thrush, may be triggered by oral sex or a change in sexual partner, could be caused by soap based products or related to a low immune system. Commonly however, women reported clinicians in general often said they simply did not know what caused BV, which women found very frustrating.

*…unfortunately every single one of them says to me*, *‘We don’t know exactly what makes it occur*, *we don’t [know]what prompts BV*, *why you get it*, *the original reason’*. *[They] can’t tell me the things that make me get it (Participant 5*, *age 38)*.

A number of women acknowledged however, that despite their frustrations they understood clinicians could only provide them with the information currently available about BV, which was very limited in terms of the cause and preventative behaviours.

#### Improved public and professional knowledge, awareness and understanding

While many women acknowledged they are seeking information that is not yet available, they still felt there needed to be increased awareness of BV both publicly and professionally. Women very clearly expressed they wanted improved knowledge and support from clinicians—in particular primary care providers—including consistency in advice, and greater acknowledgement and sensitivity around the impact of recurrent BV. At a broader level, they would also like to see raised public awareness of BV through advertising campaigns and positive and improved education in schools and the community around women’s sexual health and functioning in general. Women strongly indicated they want better treatment and preventative options so that they do not have to regularly take antibiotics. Most importantly though, women want answers–they want to know what causes BV and what they can do to prevent further episodes so that they do not have experience recurrent BV any longer. As one participant summed up:

*…I wanna know why me*, *why I get it all the time*. *I want an answer*, *I want a cure*! *(Participant 25*, *age 37)*

### Differences between Groups of Women

As part of the study we explored possible differences in the experiences of heterosexual women and WSW, single women and women in a relationship and sex industry workers and non-sex industry workers. Differences have been reported previously [10], however briefly, in relation to the data presented in this paper, we found women who worked in the sex or health industries, attended sexual health clinics or participated in the longitudinal BV study had higher levels of knowledge about BV. WSW were also more likely than heterosexual women to report working with their partner to prevent further recurrences, mainly through partner treatment or a change in sexual practices thought to have triggered BV onset or recurring symptoms.

## Discussion

In this study we found women had low levels of knowledge of BV prior to first diagnosis, commonly misdiagnosed and treated themselves for thrush in the first instance and often did not seek medical assistance for a number of weeks as a result. Women frequently reported high levels of frustration and dissatisfaction with current treatment options and clinical care management, often experiencing adverse side effects and high rates of recurrence and reporting a dislike of taking regular antibiotic treatment. Issues around clinical care management centred mainly on primary care and included inconsistency in advice, misdiagnosis and inappropriate diagnostic approaches and insensitive or dismissive attitudes lacking acknowledgment of the impact of BV on women’s lives. Women were more inclined to report positive experiences with sexual health physicians.

The majority of women had tried various self-help remedies and made lifestyle modifications in an attempt to treat symptoms or prevent further recurrences, however with little effect. Only a few women reported their self-help remedies or lifestyle modifications were successful in treating symptoms or preventing recurrences and these women were more likely to feel in control of their BV.

In a context of considerable uncertainty about the aetiology of BV, high rates of recurrence, sub-optimal treatment options, often insensitive and inconsistent clinical advice, and distressing symptoms, it is not surprising women are desperately trying all manner of self-help remedies in an attempt to prevent further BV recurrences. Past studies have found women with vaginal conditions or recurring STIs commonly use various self-help remedies to try and treat vaginal symptoms [15, 18–24] and in a recent study by Payne et al [15] researchers found 50% of women reported regularly douching to dispel vaginal odour despite being a well-publicised risk factor for BV. Previous data have shown women commonly feel dissatisfied with the efficacy of current antibiotic treatment options for BV and associated side effects and want more effective, alternative treatments [15, 24].

Contributing to their frustration and dissatisfaction with current treatment regimens for BV, is their inability to get answers from clinicians about why they are getting BV or what they can do to prevent it recurring. Previous studies have shown women commonly report dissatisfaction with the clinical care of vaginal symptoms [18, 21], with clinicians commonly regarding BV as a minor, non-serious vaginal infection [8, 9, 18, 21], not acknowledging or understanding the impact of symptoms on women’s lives [8, 10, 18] and often misdiagnosing vaginal symptoms without examination or the use of appropriate diagnostic methods [20, 31–34]. In a study by Dowd et al [23] of women presenting with vaginal symptoms in primary care, researchers found only half the women were examined vaginally when presenting with symptoms indicative of BV, most were informed they had thrush and almost all treated with antifungal medication. Further studies have found even when using recommended diagnostic methods errors in the diagnosis of BV can be high [40, 41]. While it is possible diagnostic testing of BV may prove difficult for pragmatic reasons, poor clinical attitudes are likely to only intensify the embarrassment women feel around the diagnosis and symptoms of BV and reinforce their reluctance to discuss the issue, perpetuating low levels of knowledge [20, 23] and the tendency for self-misdiagnosis and treatment among women [20, 22, 23, 42] and potentially impact on women’s likelihood to access clinical care, which in turn may delay diagnosis, reduce access to recommended treatment and increase recurrence rates.

### Strengths and Limitations

The strengths and limitations of this study have been outlined in detail in previous publications from this study [10]. Briefly, the strength of this study is the in-depth data collected on how women manage recurrent BV and their experiences of clinical care of BV—no other study we are aware of to date has specifically examined this area in such detail. A limitation of this study is that women were only recruited from an urban area and therefore the findings may not be generalizable to women living in other populations i.e. rural or regional areas or from different cultural backgrounds where beliefs about women’s vaginal conditions, sexual health and medical care may result in different perceptions.

### Future implications

Gaining a better understanding of women’s experiences and management of BV allows the opportunity to improve clinical management and diagnostic approaches. Increased understanding and acknowledgement among clinicians of the psychosocial impact of recurrent BV on women’s lives [10], consistency in advice, direct acknowledgement of areas of scientific uncertainty, use of standardized diagnostic approaches, not assuming all vaginal discharge is thrush, and not trivialising the condition, will go a long way toward addressing women’s clinical management concerns. It is also important clinicians provide evidence-based advice regarding the use of self-help remedies and lifestyle modifications given the extraordinary lengths women are taking to prevent recurrences. Despite being a well-known risk factor, it is concerning the number of women in this study and others that continue to douche to treat symptoms of BV. Women need to be informed about the scientific uncertainty around BV to assist them in understanding why treatments may not be highly effective and to hopefully reduce intensive self-help approaches which may be exacerbating recurrences. Increased public education about BV and more generally about vaginal health is also necessary to increase women’s knowledge and awareness of BV, reduce their tendency for incorrect self-diagnosis and assist in accessing timely and appropriate medical care and in protecting their reproductive health.
